# Cervical cancer prevention among long-term screening non-attendees by vaginal self-collected samples for hr-HPV mRNA detection

**DOI:** 10.1186/s13027-020-00280-0

**Published:** 2020-02-13

**Authors:** Avalon Ernstson, Annika Urdell, Ola Forslund, Christer Borgfeldt

**Affiliations:** 1 Department of Obstetrics & Gynecology, Skåne University Hospital, Lund University, Lund, Sweden; 2 Department of Medical Microbiology, Laboratory Medicine Region Skåne, Lund University, Lund, Sweden

**Keywords:** Cervical cancer, Human papillomavirus, Self-sampling, Long-term non-attendees

## Abstract

**Background:**

The efficacy of cervical cancer screening programs is dependent on the participation rate. To increase participation among women not attending cervical cancer screening, self-collected samples for detection of high-risk human papillomavirus (hr-HPV) may be an option.

The aims of this study were: to investigate the response rate to sending a self-collected vaginal sample for hr-HPV mRNA detection to long-term non-attendees; the compliance with follow-up among women positive for HPV in the self-sample; the prevalence of cervical dysplasia (high grade squamous intraepithelial lesion (HSIL), atypical squamous cells that cannot exclude HSIL (ASC-H) or adenocarcinoma in situ (AIS)) or cancer among the responders; as well as to explore reasons for not returning a self-sample.

**Methods:**

A vaginal self-sampling kit was sent to 6023 women aged 30–70 years who had not provided a cervical screening sample for ≥7 years in the Region of Skåne, Sweden in November and December 2017. The self-sample was analyzed by Aptima HPV mRNA assay (Hologic). All vaginal self-samples returned no later than May 31, 2018 were included in the study. Follow-up of the results was registered until January 31, 2019 with a follow-up time varying between eight to 14 months. Women positive for hr-HPV mRNA were invited for a follow-up examination. This examination consisted of a cervical sample for cytological analysis and renewed Aptima HPV mRNA testing. Two hundred thirty-five women who had not returned the self-sample were randomly selected for telephone interviews, in order to explore their reasons.

**Results:**

The response rate for the self-collected vaginal hr-HPV sample was 13.2% [(797/6023), 95% CI 12.4–14.1%] and 9.9% [(79/796), 95% CI 7.9–12.2%] were positive for hr-HPV mRNA. The prevalence of severe dysplasia or cancer in the whole group of responders was 1.3% [(10/796), 95% CI 0.6–2.3%], with a cervical cancer prevalence of 0.4% [(3/796), 95% CI 0.1–1.1%]. Only 27 women participated in the telephone interviews, no particular reason for not returning self-samples was observed.

**Conclusions:**

Self-collected vaginal hr-HPV samples increased participation in the cervical cancer screening among long-term non-attendees. The prevalence of cervical cancer was almost seven times higher for long-term non-attendees than in the organized screening population.

## Background

The implementation of nationwide screening programs has caused a reduction in cervical cancer cases in many Western countries [1, 2]. In Sweden, a more than 50% decrease in cervical cancer incidence has been seen since the introduction of an organized screening program in the late 1960s [3]. However, despite huge success in the preventive work against cervical cancer, there are still approximately 500 women being diagnosed with cervical cancer every year in Sweden [4]. The greatest risk factor is non-attendance at cervical cancer screening [5]. In 2017, the coverage level of cervical cancer screening was 82.9% in Sweden [6]. This is below the national recommended coverage level of 85% [7]. With the implementation of screening methods for detection of high-risk human papillomavirus (hr-HPV), the major causal factor of cervical dysplasia and invasive cancer [8], a vaginal hr-HPV self-sampling method performed by the woman herself at home has become an option. These self-collected samples could be one way to reach screening non-attendees and are recommended in the Swedish national guidelines [9]. However, on August 29, 2019, only seven out of 21 regions in Sweden had implemented self-collected sampling of screening non-attendees, making it important to evaluate the results of the few regions that use self-collected HPV samples [10].

Several hr-HPV tests for cervical- and self-sampling are available on the market. The hr-HPV mRNA tests have shown similar sensitivity but improved specificity compared to hr-HPV DNA tests taken from the cervix [11, 12]. For vaginal self-collected samples a meta-analysis by Arbyn et al. showed a lower sensitivity for the hr-HPV mRNA test compared to clinician-taken samples [13]. However, two other studies showed a substantial agreement between a self-collected vaginal sample and a clinician taken sample analyzed by Aptima mRNA assay [14, 15]. Asciutto et al. found a similar clinical sensitivity of self-collected samples analyzed by Aptima mRNA assay as compared to routine cytology. Recently an improvement of this self-collection approach showed a sensitivity of 95.3% for severe dysplasia [16, 17].

Previous studies have shown response rates for self-sampling among non-attendees to be between 15 and 58% [18–24]. Reasons for declining a vaginal hr-HPV self-sample have been explored by a few studies by sending out questionnaires. Common reasons for not returning self-samples have been; opportunistic screening outside the screening program, preference for the regular screening procedure, insecurity about how to collect the specimen themselves, the belief that screening is unnecessary, pregnancy and previous hysterectomy [20, 25–27]. To our knowledge, no previous study has used a telephone interview to explore reasons for not returning a self-sample.

The first aim of this study was to investigate the response rate of a free of charge self-collected vaginal hr-HPV sample sent to women who had not attended organized cervical cancer screening for ≥7 years. The second aim was to study the compliance with follow-up among women positive for hr-HPV in the self-collected vaginal sample. The third aim was to analyze the prevalence of severe cervical dysplasia (high grade squamous intraepithelial lesion (HSIL), atypical squamous cells that cannot exclude HSIL (ASC-H) or adenocarcinoma in situ (AIS)) or cancer among the responders. The fourth aim was to explore, by telephone interviews, the reasons for not returning a self-collected vaginal hr-HPV sample.

## Methods

### Participants

Six thousand and twenty-three women in the county of Skåne, aged 30–70 years, who had not given a cervical smear for ≥7 years were identified through the southern regional cervical cancer screening registry. The registry contains information on all obtained smears, whether organized or spontaneously taken, in the region. Kits were sent out to randomly selected women who had not attended cervical cancer screening for seven years or more in the region of Skåne. The age range was chosen according to the ages that are tested with hr-HPV testing in the regular screening program in Sweden.

### The Swedish national cervical screening program

In Sweden, all women 23–64 years old are invited to cervical cancer screening free of charge. Women aged 23–29 years old are invited every third year for primary screening with cytology. Women aged 30–49 years are invited every third year for primary screening with HPV testing and women aged 50–64 years are invited every fifth year (every seventh year in some regions in Sweden) for primary screening with HPV testing (if no HPV test is taken at 64 years old, yearly invitations are sent up to the age of 70 years). Testing using HPV as the primary screening method has been recommended since 2015 in the Swedish national guidelines and was implemented in the region of Skåne in January 2017. As a strategy to reach the non-attending women, the national guidelines of Sweden recommend giving a telephone reminder to women with no cervical cancer screening > 3 years since the last regular screening invitation. The guidelines also recommend offering self-collected vaginal hr-HPV samples to women with no cervical cancer screening registered > 4 years since the last regular screening invitation [9, 28].

### Study procedure

During November and December 2017, the identified women were offered a free of charge hr-HPV self-sampling test. The self-sampling parcel contained; 1) information about hr-HPV infection and written instructions in Swedish as well as descriptive illustrations showing how to perform the self-sampling, 2) one Aptima Multitest Swab and a tube prefilled with 2.9 ml Aptima Multitest Swab Transport Media (Hologic Inc., Marlborough, MA, USA), 3) one cylindrical container for transportation of the self-sample, 4) pre-printed labels with each woman’s social security number to mark the test, and 5) one prepaid padded return envelope. The self-sample was collected by placing a cotton swab 5 cm up into the vagina and rotating it, thereafter the cotton swab was put into the tube containing transport media. The women were asked to carefully check that the social security number was correct before affixing the pre-printed labels onto the test. The department of Laboratory Medicine, Region Skåne, Lund received the self-samples and conducted the human papillomavirus (HPV) analyses. All vaginal self-samples returned no later than May 31, 2018 were included in the study. No reminder was sent out if the kit was not returned. Follow-up of the results was registered until January 31, 2019 with a follow-up time varying between eight to 14 months. The self-samples were analyzed by Aptima HPV mRNA assay (Hologic Inc) on a Panther instrument, according to the manufacturer’s instructions. The assay detects HPV mRNA from 14 h-HPV types [16, 18, 31, 33, 35, 39, 45, 51, 52, 56, 58, 59, 66 and 68].

### Follow-up algorithm

Women with a negative HPV test result were informed that no hr-HPV types had been found and that no further testing was needed. This was done with an automatically generated letter from the department of Laboratory Medicine. Women with invalid test results were informed via a letter and were asked to make an appointment with a midwife to take a cervical HPV sample. Women with a positive HPV test result received a letter from the nearest midwife health station with information about the presence of hr-HPV and an invitation to attend a clinical follow-up examination with the midwife within three months. The follow-up examination included providing a cervical sample for cytological analysis and Aptima HPV mRNA testing. A reminder letter was sent if the woman did not attend her midwife appointment. One year after the self-sampling kit was sent out (November 2018), women with a positive hr-HPV test result that had still not attended their midwife appointment were reminded to do so by telephone. If the woman could not be reached by telephone a second reminder letter was sent informing the woman about the necessity for a follow-up examination. In case of abnormal test results at the follow-up examination, the women were managed according to regional guidelines [28]. Since January 2017, the terms HSIL and low grade squamous intraepithelial lesion (LSIL) have been used instead of cervical intraepithelial neoplasia (CIN) I-III in Sweden for classification of cytological and histological findings [9]. In this study, the worst cytology/histology diagnosis was used in case of several findings.

### Telephone interviews

To investigate reasons for not returning a self-collected vaginal hr-HPV sample, telephone interviews were conducted in October 2018. Two hundred thirty-five women were randomly selected from the self-sample non-responders group and called on the number given to their care provider. If not reached, every woman was called a total of three times at different times of day. Women who were successfully reached were informed about the study, the voluntary participation, and that all answers were handled confidentially. If the woman agreed to participate the first question asked was “Have you received a vaginal hr-HPV self-sampling invitation?” If she had not, no further questions were asked. If the invitation had been received an open second question was asked “Why did you not perform the self-sampling?” The answers were classified into five different categories, namely: emotional/attitude, practical, physical, needless, or other.

### Statistical analyses

Statistical comparisons were based on the binomial distribution and 95% confidence intervals (CI) were calculated. Microsoft® Excel, Version 15.30 was used on a Mac computer for the statistical analyses.

### Ethical approval

The study was approved by the Regional Ethical Review Board, Lund (DNR 2013/390). Returning the self-sample was defined as the woman’s consent to participate in the study.

## Results

**Fig. 1:**
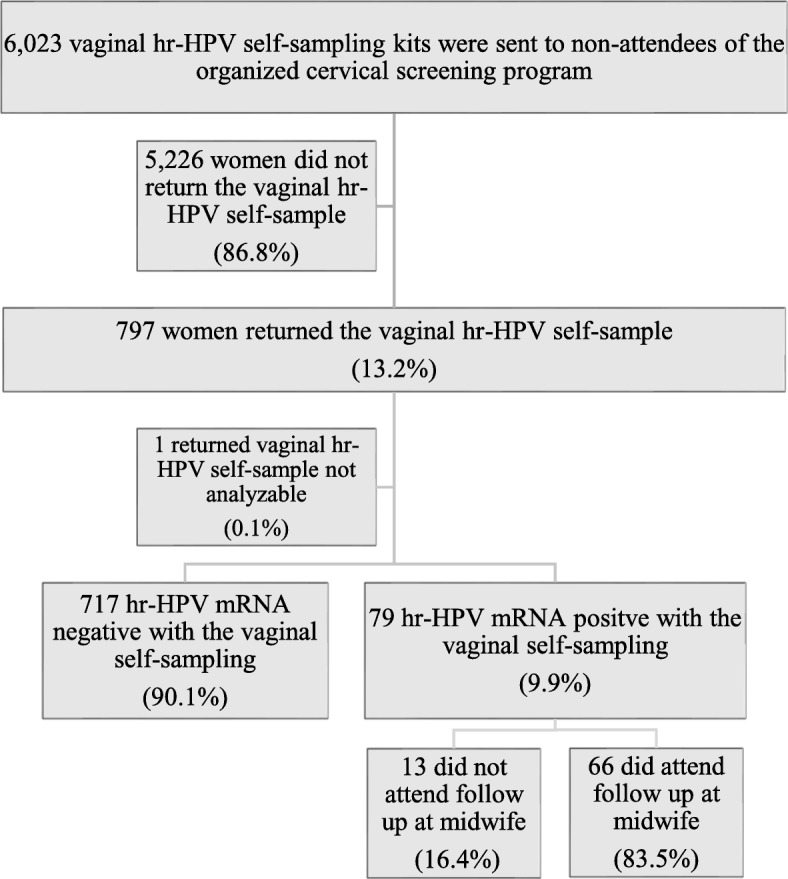
Flow-chart showing study population for invitation to self-collected vaginal hr-HPV sampling. Hr-HPV: High risk Human Papillomavirus

**Fig. 2:**
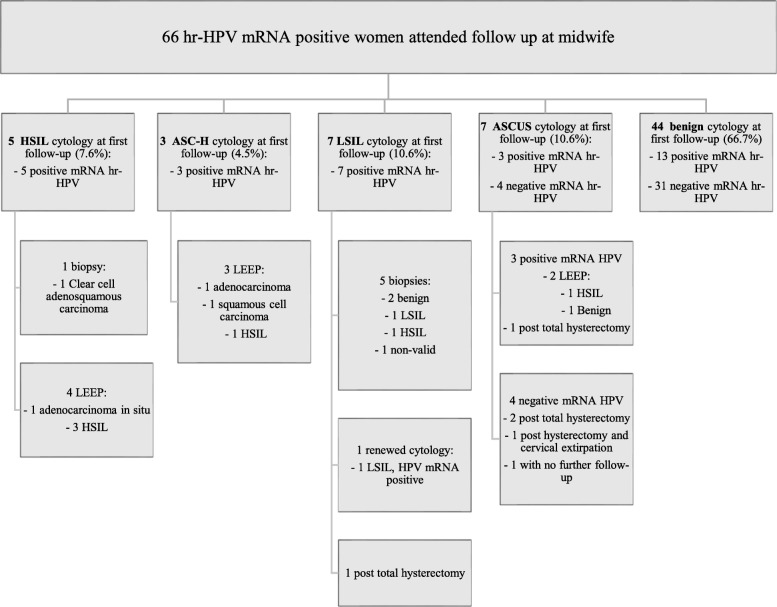
Flow-chart showing results of follow-up at midwife for the 66 women who were hr-HPV mRNA positive in the self-collected vaginal hr-HPV sample. Hr-HPV: High risk Human Papillomavirus. LEEP: Loop electrosurgical excision procedure. HSIL: High grade squamous intraepithelial lesion. ASC-H: Atypical squamous cells cannot exclude HSIL. LSIL: Low grade squamous intraepithelial lesion. ASCUS: Atypical squamous cells of undetermined significance

The response rate of the self-sample was 13.2% [(797/6023), 95% CI 12.4–14.1%] (Fig. 1). One returned self-sample could not be analyzed due to insufficient sample material, leaving 796 self-samples for hr-HPV mRNA analysis. The mean age of the women who submitted their self-samples was 61.2 years (range 33–71 years – the women 71 years of age turned 71 years during the follow-up time). Response rate and prevalence of hr-HPV mRNA stratified by age groups are shown in Table 1. Hr-HPV mRNA was detected among 9.9% [(79/796), 95% CI 7.9–12.2%] of the self-samples (mean age 60.8 years, range 37–70). Out of the women with detection of hr-HPV mRNA in the self-sample 83.5% [(66/79), 95% CI 73.5–90.9%] attended the midwife follow-up examination where 33.3% [(22/66), 95% CI 22.2–46.0%] presented with dysplasia at cytology, and 12.1% [(8/66), 95% CI 5.4–22.5%] with severe dysplasia (Fig. 2). The rate of histologically confirmed severe dysplasia or cancer in the whole group of responders was 1.3% [(10/796), 95% CI 0.6–2.3%], 0.4% [(3/796), 95% CI 0.1–1.1%] were diagnosed with cervical cancer (Fig. 2). The ten women with severe dysplasia or cancer had no registered cervical smear in the registers of the Region of Skåne for the last 16 years or more. Of the women with a positive hr-HPV self-sample, 16.5% [(13/79), 95% CI 9.1–26.5%] did not attend the follow-up examination despite receiving an invitation letter and thereafter a reminder letter. Among these, one woman was reached for a telephone reminder, the other 12 received a second reminder letter. On January 31, 2019, one of the 13 women had given a cervical smear with benign cytology and negative hr-HPV.

**Table 1:** Response rate by age groups and prevalence of hr-HPV mRNA stratified by age groups

Age group	Hr-HPV positive samples		Hr-HPV negative samples		Total HPV-tested samples		HPV prevalence
	n	%	n	%	n	%	%
≤39	1	1.3	11	1.5	12	1.5	8.3
40–49	12	15.2	73	10.2	85	10.7	14.1
50–59	16	20.3	170	23.7	186	23.4	8.6
60–69	40	50.6	375	52.3	415	52.1	9.6
70 and 71	10	12.7	88	12.3	98	12.3	10.2
Total	79	100	717	100	796	100	9.9

*Hr-HPV* High risk Human Papillomavirus

### Cervical cancer cases

Cytology of two of the women with cervical cancer showed they had ASC-H. One of these was diagnosed with squamous cell carcinoma FIGO (The International Federation of Gynecology and Obstetrics) stage IA1 and the other woman was diagnosed with adenocarcinoma FIGO stage IIB. Cytology of the third woman with cervical cancer showed she had HSIL, and she was diagnosed with clear cell adenosquamous carcinoma FIGO stage IIA1. According to our available registers in the Region of Skåne, none of the women diagnosed with cervical cancer had a registered cervical smear in the last 20 years and their last cervical smears had benign cytology.

### Telephone interview

**Fig. 3:**
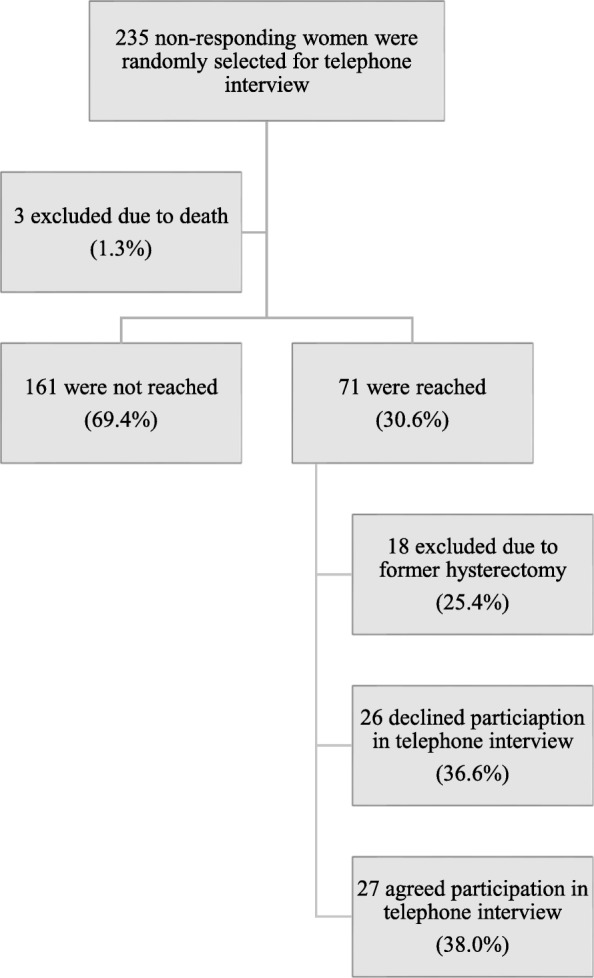
Flow-chart of the study population for telephone interviews. Two hundred and thirty-five women were randomly selected from the group of 5226 non-responders of the self-collected vaginal hr-HPV sample

Among the 235 non-responding women randomly selected for telephone interview, 1.3% [(3/235), 95% CI 0.3–36.9%] were excluded due to death from unknown causes during the follow-up period. Out of the reminding 232 women, 30.6% [(71/232), 95% CI 24.7–37.0%] of the women were reached. Among these, 25.4% [(18/71), 95% CI 15.8–37.1%] were excluded due to former hysterectomy and 38.0% [(27/71), 95% CI 26.8–50.3%] agreed to participate in the telephone interview (Fig. 3). Among the answers to the question of why the woman did not perform the self-sampling, “practical reasons” was the answer given by 40.7% [(11/27), 95% CI 24.5–59.3%], “other reasons” was given by 33.3% [(9/27), 95% CI 16.5–54.0%] and “emotional/attitude reasons” by 25.9% [(7/27), 95% CI 11.1–46.3%] (Table 2).

**Table 2:** Table showing answers to the question “Why did you not perform the self-sampling?” among women who did not respond to the vaginal hr-HPV self-sampling and agreed participation in telephone interview

Reasons for not taking or returning a vaginal hr-HPV self-sample	Women (n)	Percentage (%)
Emotional/attitude		
Fear of discomfort	0	0
Feeling healthy	0	0
Phobia/fear of cancer	3	11.1
Ignorance of cervical cancer screening	3	11.1
Insecurity around new test method	1	3.7
Total emotional/attitude reasons	**7**	**25.9**
Practical		
Lack of time	1	3.7
Forgot	5	18.5
Laziness	3	11.1
Too complicated instructions	2	7.4
Total practical reasons	**11**	**40.7**
Physical		
Movement disability restricting self-sampling	0	0
Total physical reasons	**0**	**0**
Needless		
Recent testing elsewhere	0	0
Total needless reasons	**0**	**0**
Other		
Other diseases prioritized	3	11.1
Did not received a self-sampling kit	6	22.2
Total other reasons	**9**	**33.3**
	27	100

*Hr-HPV* High risk Human Papillomavirus

## Discussion

Long-term non-attendees of the organized cervical cancer screening program are at greater risk of cervical cancer [5, 29]. Our study found that 13% of 6023 women with no cervical smear for ≥7 years returned a self-collected vaginal sample for hr-HPV testing. The prevalence of severe dysplasia and cancer was 1.3% and the prevalence of solely cervical cancer was 0.4%, which was several times higher than expected.

The histologically confirmed prevalence of severe dysplasia and cancer of 1.3% in this study corresponded to the results of 0–3.1% found in other studies using self-sampling among women with no cervical sample for 3–9 years [18, 20–23, 30, 31]. This was also in line with the prevalence of severe dysplasia or cancer of 1.4% found in the organized cervical cancer screening program in Sweden 2016 [32]. However, the prevalence of cervical cancer (0.4%) was found to be almost seven times higher in our study compared to the cervical cancer prevalence in the organized screening in Sweden 2016 (cervical cancer prevalence 2016 = 0.06%) [32]. Considering the number of self-samples received in our study, no cases of cervical cancer should have been found. Our results indicate that non-attendees of cervical cancer screening have an increased risk of cervical cancer development, a conclusion which corresponds to the findings of a systematic-review and meta-analysis by Spence et al. [29]. It is also in agreement with a Swedish study by Andrae et al. where 64% of all cervical cancer cases and 83% of advanced cases of cancer were found in women with no cervical smear taken during the recommended screening interval [5]. However, it is important to emphasize that the women diagnosed with cervical cancer in this study had no registered cervical smear in the registers in Region of Skåne in the last 20 years. This period is more than four times the length of the recommended screening interval. A similar trend with a long interval (≥16 years) since the last cervical smear could also be seen for the women with severe dysplasia. This was the first time these women had been offered a self-sampling test. Our study shows that self-samples can reach women who have not attended cervical cancer screening for a very long time. However, we need to reach the non-attending women earlier to prevent early dysplasia from developing into severe dysplasia or cancer. Thus, offering a self-sampling test after 7–9 years of non-attendance as recommended in the Swedish National guidelines could have potential for decreasing the incidence of cervical cancer among non-attendees.

The prevalence of hr-HPV mRNA in the genital tract was 10% in this study. This result was similar to that of Des Marais et al. who found an hr-HPV prevalence of 12.4% using self-collected samples analyzed by Aptima mRNA assay among women with no cervical sample in the past four years in North Carolina, USA [14]. Compared to women ≥30 years in the county of Skåne who had attended cervical cancer screening regularly, our hr-HPV prevalence was slightly higher (7% in regularly screened women vs. 10% in this study) [33]. It is also of interest that 29.5% (13/44) of women with benign cytology at the follow-up were positive for cervical hr-HPV mRNA, compared to 4% hr-HPV mRNA positivity among women aged 40–42 years with normal cytology in the regular screening program [33]. Apart from indicating that long-term non-attendees are a risk population, this could also indicate that the women participating in self-sampling may categorize themselves as individuals at high risk of HPV infection and therefore they chose to participate. However, our data cannot provide any further information on this matter.

Arbyn et al. recently published a meta-analysis investigating strategies to reach under-screened or not screened women by offering self-samples. The meta-analysis showed a response rate of between 6.4 to 34.0% (average 19.2%) when a self-sample was mailed to a woman’s home [13]. In our study, the response rate for self-collected vaginal hr-HPV samples was 13%. All self-samples returned until May 31, 2018 (approximately six months after the self-sampling offer) were included in our study. After this date, an additional of 217 women returned their self-collected sample, these samples were not included in the study. Szwarewski et al. found a response rate for self-samples returned within six months among persistent non-responders of 6.4% (10.2% including women who chose clinician cervical sampling instead) [34] and Stenvall et al. found a response rate for self-samples returned within five months among women with no cervical sample for ≥6 years of 32% [35]. Furthermore, Sancho-Garnier et al. found a response rate for self-samples of 18.3% of which 13.3% of the women had never taken a cervical smear according to the records [36]. These data show a large variation in response rate for self-collected samples among non-attendees. Previous studies have shown that reminders could increase the self-sampling participation rates among non-attendees [18, 22], however, no reminder was sent in this study. In 2017, the coverage of the cervical cancer screening program in Skåne was 81.5% [6], an addition of 13% among all the former non-attendees in the entire county of Skåne generates a total coverage level of nearly 84%. This is a small but important step in a desirable direction. The mean age of the women returning the self-collected sample was 61.6 years in this study. Comparing to a study by Darlin et al. conducted in the same geographical area as our study, Darlin et al. found a mean age of women returning a self-collected HPV sample of 51 years (range 32–64 years) [18]. Although in another more recent study conducted in the same geographical area we studied the response rate of a self-collected HPV sample sent to screening non-attending women 69–70 years old and found a high response rate of 43.3% [37]. This could indicate that older women in this area have a positive attitude towards self-sampling.

Compliance with follow-up is essential if self-sampling is to be used. In this study, 83.5% of women with a HPV positive self-collected sample attended the midwife follow-up examination. This is similar to a recent meta-analysis by Arbyn et al. where the average participation rate for follow-up was 80.6% [13]. Compared to other studies conducted in Sweden among women who had provided no cervical sample for ≥6–9 years the compliance with follow-up varied between 70 and 100% [18, 20, 21, 35, 38].

Our study is the first, as far as we know, using telephone interviews to explore reasons for not returning a vaginal hr-HPV self-sample among long-term non-attendees of the organized cervical cancer screening program. The participation rate of 12% in the telephone interviews was below our expectations, but the results should be interpreted with some caution because of the small sample size. Previous studies using questionnaires have reached participation rates of 3.4–38% [19, 20, 25]. The most common reasons for not taking part in the organized cervical cancer screening in Sweden are “uncomfortable with vaginal examination”, “feel healthy”, “lack of time” and “experience of unfriendly health workers” [18]. In our study, the six most common reasons for not returning a vaginal hr-HPV self-sample were “did not receive a self-sampling kit”, “forgot”, “phobia/fear of cancer”, “ignorance of cervical cancer screening”, “laziness” and “other diseases prioritized”. A common reason for non-attendance in both settings was “forgot”. It is also noteworthy that 22% of the women answered that they did not receive their self-sampling kit. An individual telephone dialog would provide the opportunity to ensure that all women have received the self-sampling kit, and would also serve as a reminder and give a chance to motivate the women based on their individual reasons. This strategy is also in line with the recommendations in the National Guidelines [39], but it is time-consuming for health personnel.

This study was performed in a setting of non-attendees of a current population-based cervical cancer screening program, which is a strength of the study. Furthermore, only one self-collected sample was invalid for analysis in the final compilation of results. Notably, during the analytical process we experienced problems with invalid samples (in one batch 25% (110/434) of the samples was invalid), but re-analysis of ¼ diluted samples (1 ml sample were transferred to 2.9 ml) rendered such samples as valid. However, for future studies of self-collected samples analyzed for HPV mRNA by the Aptima-system we will instead add a pre-heating step of samples, in order to reduce the proportion of invalid samples [17]. One limitation of the study is that women with a previous total hysterectomy, which is a criterion of exclusion from cervical cancer screening [39], were not excluded from the study. According to figures from the Swedish National Patient registry, 5–6% of Swedish women in the age group 40–60 years have had a hysterectomy with removal of the cervix [40]. This may have affected the number of self-samples submitted and shows that improvements are needed to prevent women who have no cervix from being incorrectly invited to attend cervical cancer screening. In this study, women in the Region of Skåne who had provided no cervical sample for ≥7 years were included. However, we do not have information about whether the women had provided a more recent cervical sample in a different region of Sweden or in another country. The participation rate for returning the self-sample is rather low in this study, and no reminder was sent out. A reminder may increase the participation rate, which is why we recommend a reminder letter or a phone call in future studies. The participation rate in the telephone interviews was also low, partly because of invalid telephone numbers which might have been a consequence of the switch from land-line to mobile phone systems currently taking place in Sweden.

## Conclusions

In conclusion, offering self-collected vaginal samples for hr-HPV mRNA testing was a promising strategy to increase participation in the cervical cancer screening among long-term non-attendees. The compliance with follow-up was 83.5%, which is a good level, comparable to other studies. The prevalence of cervical cancer was almost seven times higher among long-term non-attendees than in the organized screening population. No particular reason for not returning self-samples was noted from the conducted telephone interviews.

## Data Availability

The datasets used and analyzed during the current study are available from the corresponding author on reasonable request.

## Abbreviations

AIS: Adenocarcinoma in situ
ASC-H: Atypical squamous cells that cannot exclude HSIL
ASCUS: Atypical squamous cells of undetermined significance
CI: Confidence interval
CIN: Cervical intraepithelial neoplasia
FIGO: The international federation of gynecology and obstetrics
HPV: Human papillomavirus.
Hr-HPV: High risk human papillomavirus.
HSIL: High grade squamous intraepithelial lesion.
LEEP: Loop electrosurgical excision procedure.
LSIL: Low grade squamous intraepithelial lesion.

## References

[1] Arbyn M, Raifu AO, Weiderpass E, Bray F, Anttila A (2009). Trends of cervical cancer mortality in the member states of the European Union. Eur J Cancer.

[2] Pedersen K, Fogelberg S, Thamsborg LH, Clements M, Nygard M, Kristiansen IS (2018). An overview of cervical cancer epidemiology and prevention in Scandinavia. Acta Obstet Gynecol Scand.

[3] Bergman O, Fredholm L, Hont G, Johansson E, Ljungman P, Munck-Wikland E, et al. Cancer i siffror 2018. [Cancer in figures 2018]. In Swedish. Socialstyrelsen [National Board of Health and Welfare], Cancerfonden [The Swedish Cancer Society] 2018 Article Number 2018-6-10.

[4] National Board of Health and Welfare. Statistical Database - Cancer. Stockholm: Socialstyrelsen. http://www.socialstyrelsen.se/statistik/statistikdatabas/cancer. Accessed 18 Dec 2018

[5] Andrae B, Kemetli L, Sparen P, Silfverdal L, Strander B, Ryd W (2008). Screening-preventable cervical cancer risks: evidence from a nationwide audit in Sweden. J Natl Cancer Inst.

[6] Swedish National Cervical Screening Registry. Quality indicators - Coverage per region: Nationellt Kvalitetsregister för Cervixcancerprevention. Stockholm.http://www.nkcx.se/Covr_all.htm. Accessed 18 Dec 2018.

[7] Dillner J, Strander B, Sparén P. Förebyggande av livmoderhalscancer i Sverige - Verksamhetsberättelse och Årsrapport 2018 med data till och med 2017. [Prevention of Cervical Cancer in Sweden - Activity Report and Annual Report 2018 with data up to and including 2017]. In Swedish. Nationellt Kvalitetsregister för Cervixcancerprevention [Swedish National Cervical Screening Registry]. Stockholm. 2018. Place published: http://www.nkcx.se/templates/_rsrapport_2018.pdf. Accessed 18 Dec 2018.

[8] Walboomers JM, Jacobs MV, Manos MM, Bosch FX, Kummer JA, Shah KV (1999). Human papillomavirus is a necessary cause of invasive cervical cancer worldwide. J Pathol.

[9] Regionala Cancercentrum i Samverkan. Cervixcancerprevention. Nationellt vårdprogram. [Regional Cancer Centres. Cervical cancer prevention. National care program]. In Swedish. 2019-09-23.Version 2.2. https://www.cancercentrum.se/globalassets/vara-uppdrag/prevention-tidig-upptackt/gynekologisk-cellprovskontroll/vardprogram/nationellt-vardprogram-cervixcancerprevention.pdf. Accessed 02 Jan 2019.

[10] Regionala Cancercentrum i Samverkan (RCC). Status för införandet av vårdprogrammet för livmoderhalscancerprevention. [Regional cancer centres in collaboration. Status of the introduction of the cervical cancer prevention program.] In Swedish. [updated Nov 25 2019] https://www.cancercentrum.se/samverkan/vara-uppdrag/prevention-och-tidig-upptackt/gynekologisk-cellprovskontroll/vardprogram/status-for-inforandet/. Accessed 29 Dec 2019.

[11] Arbyn M, Snijders PJ, Meijer CJ, Berkhof J, Cuschieri K, Kocjan BJ (2015). Which high-risk HPV assays fulfil criteria for use in primary cervical cancer screening?. Clin Microbiol Infect.

[12] Reid JL, Wright TC, Stoler MH, Cuzick J, Castle PE, Dockter J (2015). Human papillomavirus oncogenic mRNA testing for cervical cancer screening: baseline and longitudinal results from the CLEAR study. Am J Clin Pathol.

[13] Arbyn M, Smith SB, Temin S, Sultana F, Castle P, Collaboration on S-S (2018). Detecting cervical precancer and reaching underscreened women by using HPV testing on self samples: updated meta-analyses. BMJ.

[14] Des Marais AC, Zhao Y, Hobbs MM, Sivaraman V, Barclay L, Brewer NT (2018). Home self-collection by mail to test for human papillomavirus and sexually transmitted infections. Obstet Gynecol.

[15] Chernesky M, Jang D, Gilchrist J, Elit L, Lytwyn A, Smieja M (2014). Evaluation of a new APTIMA specimen collection and transportation kit for high-risk human papillomavirus E6/E7 messenger RNA in cervical and vaginal samples. Sex Transm Dis.

[16] Asciutto KC, Ernstson A, Forslund O, Borgfeldt C (2018). Self-sampling with HPV mRNA analyses from vagina and urine compared with cervical samples. J Clin Virol.

[17] Borgfeldt C, Forslund O (2019). Increased HPV detection by the use of a pre-heating step on vaginal self-samples analysed by Aptima HPV assay. J Virol Methods.

[18] Darlin L, Borgfeldt C, Forslund O, Henic E, Hortlund M, Dillner J (2013). Comparison of use of vaginal HPV self-sampling and offering flexible appointments as strategies to reach long-term non-attending women in organized cervical screening. J Clin Virol.

[19] Bosgraaf RP, Siebers AG, De Hullu JA, Massuger LF, Bulten J, Bekkers RL (2014). The current position and the future perspectives of cervical cancer screening. Expert Rev Anticancer Ther.

[20] Wikstrom I, Stenvall H, Wilander E (2007). Low prevalence of high-risk HPV in older women not attending organized cytological screening: a pilot study. Acta Derm Venereol.

[21] Sanner K, Wikstrom I, Strand A, Lindell M, Wilander E (2009). Self-sampling of the vaginal fluid at home combined with high-risk HPV testing. Br J Cancer.

[22] Broberg G, Gyrd-Hansen D, Miao Jonasson J, Ryd ML, Holtenman M, Milsom I (2014). Increasing participation in cervical cancer screening: offering a HPV self-test to long-term non-attendees as part of RACOMIP, a Swedish randomized controlled trial. Int J Cancer.

[23] Gok M, Heideman DAM, van Kemenade FJ, Berkhof J, Rozendaal L, Spruyt JWM (2010). HPV testing on self collected cervicovaginal lavage specimens as screening method for women who do not attend cervical screening: cohort study. BMJ.

[24] Bais AG, van Kemenade FJ, Berkhof J, Verheijen RHM, Snijders PJF, Voorhorst F (2007). Human papillomavirus testing on self-sampled cervicovaginal brushes: an effective alternative to protect nonresponders in cervical screening programs. Int J Cancer.

[25] Virtanen A, Nieminen P, Niironen M, Luostarinen T, Anttila A (2014). Self-sampling experiences among non-attendees to cervical screening. Gynecol Oncol.

[26] Bosgraaf RP, Ketelaars PJ, Verhoef VM, Massuger LF, Meijer CJ, Melchers WJ (2014). Reasons for non-attendance to cervical screening and preferences for HPV self-sampling in Dutch women. Prev Med.

[27] Nelson EJ, Maynard BR, Loux T, Fatla J, Gordon R, Arnold LD (2017). The acceptability of self-sampled screening for HPV DNA: a systematic review and meta-analysis. Sex Transm Infect.

[28] Region Skåne. Regionala riktlinjer för screening för cervixcancer. [regional guidelines for screening for cervical cancer.] in Swedish. 2017-12-20. Place Published: https://vardgivare.skane.se/siteassets/1.-vardriktlinjer/regionala-riktlinjer%2D%2D-fillistning/screening-for-cervixcancer%2D%2D-riktlinje-171220-slutversion.pdf. Accessed 02 Jan 2019.

[29] Spence AR, Goggin P, Franco EL (2007). Process of care failures in invasive cervical cancer: systematic review and meta-analysis. Prev Med.

[30] Maza M, Melendez M, Masch R, Alfaro K, Chacon A, Gonzalez E (2018). Acceptability of self-sampling and human papillomavirus testing among non-attenders of cervical cancer screening programs in El Salvador. Prev Med.

[31] Sultana F, English DR, Simpson JA, Drennan KT, Mullins R, Brotherton JML (2016). Home-based HPV self-sampling improves participation by never-screened and under-screened women: results from a large randomized trial (iPap) in Australia. Int J Cancer.

[32] Swedish National Cervical Screening Registry. Ouality indicators - Crossreference cytology - histopathology organized screening 2016: Nationellt Kvalitetsregister för Cervixcancerprevention; http://www.nkcx.se/. Accessed 06 Jan 2019.

[33] Lindroth Ylva, Borgfeldt Christer, Thorn Gunilla, Bodelsson Gunilla, Forslund Ola (2019). Population-based primary HPV mRNA cervical screening compared with cytology screening. Preventive Medicine.

[34] Szarewski A, Cadman L, Mesher D, Austin J, Ashdown-Barr L, Edwards R (2011). HPV self-sampling as an alternative strategy in non-attenders for cervical screening - a randomised controlled trial. Br J Cancer.

[35] Stenvall H, Wikstrom I, Wilander E (2007). High prevalence of oncogenic human papilloma virus in women not attending organized cytological screening. Acta Derm Venereol.

[36] Sancho-Garnier H, Tamalet C, Halfon P, Leandri FX, Le Retraite L, Djoufelkit K (2013). HPV self-sampling or the pap-smear: a randomized study among cervical screening nonattenders from lower socioeconomic groups in France. Int J Cancer.

[37] Ernstson A, Asciutto KC, Sturesson J, Noren J, Forslund O, Borgfeldt C (2019). Detection of HPV mRNA in Self-collected Vaginal Samples Among Women at 69–70 Years of Age. Anticancer Res.

[38] Broberg G, Jonasson JM, Ellis J, Gyrd-Hansen D, Anjemark B, Glantz A (2013). Increasing participation in cervical cancer screening: telephone contact with long-term non-attendees in Sweden. Results from RACOMIP, a randomized controlled trial. Int J Cancer.

[39] Regional Cancer Centres. Cervixcancerprevention. Nationellt vårdprogram och Konsekvenser av införande av Socialstyrelsens rekommendationer gällande screening juni 2015. [Cervical cancer prevention. National care program and Consequences of the introduction of the National Board of Health and Welfare's recommendations regarding screening June 2015]. In Swedish. Regionala Cancercentrum i Samverkan. 2017-01-05. https://www.cancercentrum.se/globalassets/vara-uppdrag/prevention-tidig-upptackt/gynekologisk-cellprovskontroll/vardprogram/nvp-cervixcancerprevention-170119.pdf. Accessed 18 Dec 2018.

[40] The Swedish national Quality Register of Gynecological Surgery (Gynop). Annual Reports. http://www2.gynop.se/for-kliniker/arsrapporter/. Accessed 12 Jan 2020.

